# Mental well-being and recovery in serious mental illness: associations between mental well-being and functional status in the Health Survey for England 2014

**DOI:** 10.1192/bjo.2020.46

**Published:** 2020-06-18

**Authors:** Sarah Chan, Scott Weich

**Affiliations:** School of Health and Related Research, University of Sheffield, UK; Mental Health Research Unit, School of Health and Related Research, University of Sheffield, UK

**Keywords:** Serious mental illness, recovery, mental well-being, functioning

## Abstract

**Background:**

Mental illness and mental well-being are independent but correlated dimensions of mental health. Both are associated with social functioning (in opposite directions), but it is not known whether they modify the effects of one another. New treatment targets might emerge if improving mental well-being in people with serious mental illness improved functional outcomes independent of clinical status.

**Aims:**

To describe associations between mental well-being and functioning in people classified according to mental illness status.

**Method:**

Cross-sectional data from 5485 respondents to the Health Survey for England 2014 were analysed. Mental illness status (including whether diagnosed by a professional) was by self-report and grouped into four categories, including ‘diagnosis of serious mental illness’. Mental well-being was measured using the Warwick-Edinburgh Mental Well-Being Scale, and functioning by items from the EQ-5D. Mental distress was assessed using General Health Questionnaire (GHQ-12) items. Associations were examined using moderated regression models with group membership as an interaction term.

**Results:**

Mental well-being score was associated with (higher) functioning score (*P* < 0.05). This association varied between mental illness groups, even after adjusting for age, gender, ethnicity, physical health and symptoms of mental distress (*F*(3) = 14.60, *P* < 0.001). The gradient of this association was greatest for those with diagnosed serious mental illness.

**Conclusions:**

Mental well-being was associated with higher functional status in people with mental illness, independent of the symptoms of mental distress and other confounders. The association was strongest in the diagnosed serious mental illness group, suggesting that mental well-being may be important in recovery from mental illness.

## Mental health, mental illness and mental well-being

Mental illnesses are among the main causes of disease burden globally.^1–3^ Mental health, by contrast, is described by the World Health Organization (WHO) as ‘a state of well-being’^4^ and as ‘a positive phenomenon that is more than the absence of mental illness’ by Westerhof & Keyes.^5^ The concept of mental well-being was first introduced by the WHO in 1948 and is said to occur when an individual can cope with the normal stresses of life, work productively and fulfilling, feels happy and satisfied with their life, and is able to make a contribution to his or her community.^6^ Although mental illness and mental well-being were once seen as two ends of a single continuum (from flourishing to languishing),^7^ empirical evidence now supports the view that these are two correlated dimensions.^5,8^ This is consistent with the view that it is possible to have moderate or high levels of mental well-being (including enjoying life and/or having a sense of purpose and fulfilment) despite experiencing mental illness.

Although governments and others are committed to promoting mental well-being as a means of enhancing prosperity,^9–13^ it remains unclear whether, and to what degree, promoting mental well-being can reduce the burden of mental illness and improve outcomes for people with established mental illness. An observational study by Meyer compared 30 patients over 6 weeks with different types of mental illness.^14^ Considering coping as a proxy for functioning, the association between mental well-being (measured using the Psychological Well-Being Questionnaire^15^) and functioning was statistically significant in the schizophrenia group. Current clinical guidance for managing serious mental illnesses (such as bipolar disorder and psychosis and schizophrenia) focuses on achieving symptom resolution.^16–19^ It is not yet known whether and to what extent mental well-being may be associated with, or contribute to, functional improvements in people with mental illness, and whether this is independent of symptoms. Such an association would support the importance of developing and delivering interventions to enhance mental well-being in people experiencing episodes of mental illness.

## Aims

The aim of this study was to test whether mental well-being was associated with differences in functioning in people with a self-reported history of mental illness, after adjusting for differences in symptoms of mental distress.

## Method

### Design

We undertook a cross-sectional study, based on secondary analysis of data from a large, national population-based health survey, the Health Survey for England (HSE).^20^

### Participants and procedures

HSE started in 1991 and is an annual survey of the general adult and child population living in private households in England, representative of the population of England at both national and regional levels. The primary purpose of HSE is to provide annual data for monitoring health trends and progress towards health targets, estimate prevalence of health conditions and associated risk factors, and examine socioeconomic subgroups.

Each HSE features a core set of questions on general health, chronic illness, lifestyle behaviours and social care, along with supplementary topics that change at each survey. Data from the HSE in 2014 were chosen because they included information about mental health. HSE 2014 used a multistage stratified probability sampling design, with postcode sectors forming primary sampling units. Addresses were selected from January to December 2014 and fieldwork was completed in March 2015. Of 8204 eligible households, the household response rate was 62% and represented households where at least one person was interviewed. Non-response weights were derived by the survey team (using logistic regression) to reduce bias from non-response within households.

Questions on mental illness experience and treatment were posed verbally in an interview during the nurse visit, whereas mental well-being and functioning measures were collected via self-completed questionnaires. Of 8077 adult respondents to HSE 2014 aged 16 and over, 7441 (92.1%) returned the self-completed questionnaires and 5491 (68.0%) took part in the nurse visit. Of the respondents who made up the adult sample of the HSE 2014, 5485 (68.0%) completed the section on mental health. These 5485 respondents were subsequently used as the sample population for this study. Complete data from mental well-being and functioning measures were available for 4943 respondents (61.2% of the total adult sample) and 5053 (62.6%), respectively. Of the sample, 4862 (60.2%) provided data on all three items of interest.

## Measures

### Mental illness groups

Mental illness groups were defined according to self-reported history of mental illness. Respondents were asked whether they had experienced or were diagnosed with any mental health conditions using a list of 17 conditions. If the response was ‘yes’ to any of these, the interviewer proceeded to ask if a diagnosis had ever been given or confirmed by a doctor, psychiatrist or other professional. Serious mental illness was defined as a reported diagnosis (i.e. one that had been confirmed by a professional) of bipolar disorder, eating disorder, nervous breakdown, personality disorder, psychosis or schizophrenia. History of mental illness was classified as follows: (a) no mental illness; (b) mental illness experienced, but not diagnosed by a professional; (c) diagnosed mental illness other than serious mental illness; (d) diagnosis of serious mental illness.

### Mental well-being

The Warwick-Edinburgh Mental Well-Being Scale (WEMWBS)^21^ was administered by self-completed questionnaire. WEMWBS comprises 14 items covering psychological functioning, and cognitive-evaluative and affective-emotional aspects of well-being.^21^ All 14 items are worded positively and responses are recorded using a Likert scale.^22^ An example is: ‘I've been feeling optimistic about the future’, with responses coded from one for ‘none of the time’ to five for ‘all of the time’. Although WEMWBS includes hedonic and eudemonic items, principal component analysis only extracted one component and hence total WEMWBS score was used, derived as the sum of item responses (minimum possible score 14, maximum 70).

### Functioning

As there was no questionnaire set within HSE 2014 dedicated to measuring functioning, this was derived from responses to EQ-5D items.^23^ EQ-5D is a self-completed questionnaire that asks about general health and functioning using items covering mobility, self-care, usual activities (i.e. work, study, housework, family and leisure activities that the respondent would normally undertake), pain or discomfort, and anxiety and depression.^24^ We summed scores for the mobility, self-care and usual activities items. A response was recorded for each item using a Likert scale, with 1 representing the least problems or impediment and 3 representing extreme inability. We included only the mobility, self-care and usual activities items; the other two EQ-5D items were excluded as ‘anxiety’ is closely related to mental health and ‘pain’ is not a measure of functioning.

Principal components analysis using data from the three included EQ-5D items extracted one component, with similar weightings for all three items. We conducted further exploratory principal components analysis to include employment status.^25^ The factor loading for employment status was modest, and hence this variable was not included in the derivation of functioning scores. Consequently, functioning scores were derived by summing the mobility, self-care and usual activities item scores.

### Symptoms of mental distress

Symptoms of mental distress were ascertained using the General Health Questionnaire (GHQ-12).^26^ GHQ-12 is a self-report questionnaire with excellent psychometric properties, commonly used to screen for psychiatric disorders in non-clinical settings.^26^ It comprises 12 items, 6 worded positively and 6 worded negatively. Responses for each item are recorded using a Likert scale. The direction of scores for each item is such that a higher score indicates more severe symptoms. An example item is ‘Have you felt constantly under strain?’, with responses coded as 1 for ‘better than usual’ to 4 for ‘much less than usual’. Previous psychometric research has shown that GHQ-12 scores are best described by two factors, namely symptoms of mental distress and positive mental health.^27^ Six items were found to load onto the former factor. To avoid conflating our measures of mental well-being and mental distress, only the six negatively worded items were used to derive the mental distress score in this study, which was taken as the sum of scores on these items. The items were: ‘Have you recently (1) lost much sleep over worry; (2) felt constantly under strain; (3) felt you couldn't overcome your difficulties; (4) been feeling unhappy or depressed; (5) been losing confidence in yourself; and (6) been thinking of yourself as a worthless person?’.

### Potential confounders

Other potential confounders of the association between mental well-being and functioning were age,^28–30^ gender,^31^ ethnicity,^32–36^ long-standing illness^37–39^ and income. The HSE survey asks about the presence of long-standing illnesses, up to a maximum of six conditions. A summary physical illness variable was derived by summing the number of long-standing illness categories that respondents identified, excluding ‘mental disorders’. Equivalised household income was derived using McClements scores to account for household composition.

### Statistical analysis

Descriptive statistics regarding respondent characteristics and other variables of interest were reported by mental illness group. Preliminary one-way analysis of variance (ANOVA) was conducted to test for significant differences in WEMWBS score between mental illness groups. The median WEMWBS score of the group with no mental illness was calculated and used to compare the proportion of people within the serious mental illness group above this level.

Moderated regression analyses were used to explore whether, and to what extent, the association between mental well-being and functioning scores varied between mental illness groups.^40,41^ The moderator was represented as an interaction term for mental illness group × mental well-being (referred to as group × well-being) in the analyses. The size and statistical significance of these interaction terms were thus estimated.

Three models were developed in which functioning was the outcome: (a) model excluding interaction terms; (b) model including the interaction term for group × well-being; (c) model including group × well-being interaction and covariates. Where interactions in model 2 were not statistically significant, this was omitted from the final model.

Predicted functioning scores were derived from the regression coefficients (*B*) for each exposure group. The gradient for the slope (*m*) of each group was derived through algebraic manipulation by arranging the equation into the form of *y* = *mx* + *C*, where *y* is the dependent variable, functioning score; and *x* is the independent variable, WEMWBS score. All regression models used the group with self-reported history of ‘no mental illness’ as the reference.

To aid interpretation, results of models 2 and 3 were represented visually by means of a plot of functioning score against WEMWBS score. For both graphs, ‘low’ and ‘high’ WEMWBS scores were calculated as 1 s.d. below and above the mean for the study sample, respectively. As model 3 included covariates that are continuous variables, the graph for this model was plotted using centred variables to ensure that the value ‘zero’ is meaningful for these variables.^40^ Centring was done by subtracting the variable's sample mean from its original value.

The effect size for the interaction term (group × well-being) was calculated using Cohen's *f*^2^ statistic, which calculates the ratio of variance explained by the interaction term alone to the unexplained variance in the final model. Cohen's *f*^2^ is expressed mathematically as,
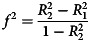
where *R*_1_^2^ is the variance explained by the model excluding the term of interest, and *R*_2_^2^ is the variance explained by the model including the term of interest. In general, a larger *f*^2^ denotes a larger effect size and vice versa.^40^ It has been suggested that the values of 0.10, 0.25, and 0.40 represent small, medium, and large effect sizes, respectively.^42^

Analyses were performed using SPSS Version 24 for Macintosh. A published SPSS syntax for an ANCOVA version of two-way interaction with a categorical moderator was used in constructing and executing the regression models.^40^

### Ethical considerations

This study involved the use of secondary data from the HSE 2014. Ethics clearance was sought and received from the Ethics Review Committee of the School of Health and Related Research at the University of Sheffield. The data-set was supplied by the UK Data Service who granted permission to use the data in its anonymised form for the purposes of this study.

## Results

Descriptive statistics for the participants (*n* = 5485) are presented in Table 1. Among the participants, 3011 (54.9%) had no mental illness, 935 (17.0%) reported mental illness without a formal diagnosis, 1298 (23.7%) had a diagnosed mental illness that was not classed as serious mental illness and 241 (4.4%) were classified as having a diagnosis of serious mental illness.

**Table 1:** Summary table of socio-demographic characteristics by exposure group^a^

Exposure group	No mental illness group (*n* = 3011)	Mental illness reported without diagnosis group (*n* = 935)	Diagnosed mental illness other than serious mental illness group (*n* = 1298)	Diagnosed serious mental illness group (*n* = 241)
Age, years: mean (s.d.)	51.9 (19.0)	51.3 (18.8)	50.4 (16.2)	51.5 (16.5)
Gender, *n* (%)				
Male	1547 (51.4)	402 (43.0)	403 (31.0)	83 (34.4)
Education, *n* (%)				
Degree or equivalent	801 (26.7)	238 (25.5)	327 (25.2)	45 (18.7)
Higher education below degree	797 (26.5)	262 (28.0)	357 (27.5)	62 (25.7)
GCE O level or equivalent	669 (22.3)	228 (24.4)	336 (25.9)	57 (23.7)
None	738 (24.4)	207 (22.1)	278 (21.4)	77 (32.0)
Ethnicity, *n* (%)				
White	2641 (87.7)	844 (90.5)	1241 (95.6)	229 (95.0)
Black	85 (2.8)	19 (2.0)	12 (0.9)	2 (0.8)
Asian	223 (7.4)	50 (5.4)	32 (2.5)	9 (3.7)
Other	61 (2.0)	20 (2.2)	13 (1.0)	1 (0.4)
Marital status, *n* (%)				
Single	501 (16.7)	168 (18.0)	223 (17.2)	50 (20.7)
Married or cohabiting	2038 (67.7)	569 (60.8)	801 (61.8)	122 (50.6)
Separated, divorced or widowed	470 (15.6)	198 (21.2)	274 (21.2)	69 (28.6)
Equivalised income, £: median (IQR)	27 431.2 (27 666.9)	26 702.7 (26 390.4)	22 783.5 (22 023.5)	19 500 (20 930.7)
Vehicle, *n* (%)				
Car or van	2551 (84.7)	759 (81.2)	1042 (80.3)	162 (67.2)
Housing tenure, *n* (%)				
Owner-occupier	2218 (73.8)	634 (67.8)	849 (65.4)	122 (50.6)
Household type, *n* (%)				
Living alone	507 (16.8)	208 (22.2)	254 (19.5)	66 (27.4)
Number of categories of long-lasting illness (excluding mental illness), mean (s.d.)	0.62 (1.01)	0.79 (1.07)	0.90 (1.14)	1.16 (1.23)
Number of respondents reporting 3 or more categories of long-lasting illness (excluding mental illness), *n* (%)	189 (6.3)	79 (8.4)	147 (11.3)	41 (17.0)

IQR, interquartile range.

a. The sample size was different for each set of results as the number of participants completing the measures concerned varied slightly.

Mental illness groups appeared similar in age, gender and ethnicity, but dissimilar in other sociodemographic characteristics. The group with a diagnosis of serious mental illness were less likely to report completing higher education, being married, access to a personal vehicle and owning their home. They also had lower equivalised incomes, were more likely to live alone and reported more long-standing physical illnesses (Table 1). The mean number of reported long-standing physical illnesses across all groups was 0.74 (s.d. = 0.07, median 0). The serious mental illness group had the highest proportion of respondents reporting three or more such illnesses. Using the median well-being score of the group with no mental illness (median 53) as a cut-off for a ‘good’ well-being score, the number of individuals within the serious mental illness group with a score above or equal to this was 56 (23.2%).

### Associations between mental well-being and mental illness

The mean WEMWBS score were 52.9 (95% CI 52.6 to 53.2) for the group with no mental illness, 50.3 (95% CI 49.8 to 50.8) for the group experiencing mental illness, 47.5 (95% CI 47.0 to 48.0) for the group with non-serious mental illness mental illness and 42.7 (95% CI 41.3 to 44.1) for the group with a diagnosis of serious mental illness. Statistically significant differences between groups were found for WEMWBS score (*F*(3) = 181.17, *P* < 0.001), and group means were different from one another, to a statistically significant degree.

### Associations between mental well-being and functioning scores

**Fig. 1:**
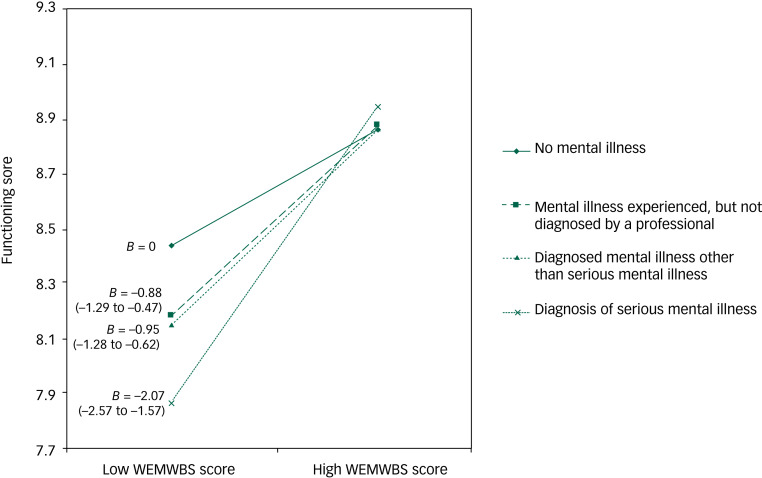
Gradients of association between mental well-being and functioning scores by mental illness group, before adjustment for covariates (model 2).

Model 1 (excluding interaction terms) showed statistically significant (*P* < 0.01) differences in functioning scores between mental illness groups (Table 2). In model 2, the group × well-being interaction was statistically significant (*F*(3) = 19.15, *P* < 0.001) (Table 2); consequently, there were statistically significant differences between the gradients shown in Fig. 1. Using adjusted *R*^2^ values from models 1 and 2, Cohen's *f*^2^ for the moderating effect of mental illness group on the association between mental well-being and functioning before including covariates was 0.011.

*B* (95% CI) labelled is the regression coefficient.

**Table 2:** Results of models showing regression for association with functioning scores (model 1), including the group × well-being interaction term without covariates (model 2) and with covariates (model 3)

Parameter	Model 1		Model 2		Model 3	
	*B* (95% CI)	*P*	*B* (95% CI)	*P*	*B* (95% CI)	*P*
Intercept	6.97 (6.82 to 7.12)	–	7.48 (7.27 to 7.69)	–	7.96 (7.76 to 8.26)	–
WEMWBS score	0.03 (0.03 to 0.04)	<0.001	0.02 (0.02 to 0.03)	<0.001	0.02 (0.01 to 0.02)	<0.001
Groups						
No mental illness	0	–	0	–	0	–
Mental illness (not diagnosed)	−0.12 (−0.18 to −0.05)	0.001	−0.88 (−1.29 to −0.47)	<0.001	−0.54 (−0.90 to −0.18)	0.003
Diagnosed mental illness (not serious mental illness)	−0.12 (−0.18 to −0.06)	<0.001	−0.95 (−1.28 to −0.62)	<0.001	−0.58 (−0.87 to −0.28)	<0.001
Diagnosed serious mental illness	−0.42 (−0.54 to −0.30)	<0.001	−2.07 (−2.57 to −1.57)	<0.001	−1.67 (−2.11 to −1.23)	<0.001
Interaction terms						
No mental illness × WEMWBS	–	–	–	–	0	−
Mental illness (not diagnosed) × WEMWBS score	–	–	0.02 (0.01 to 0.02)	<0.001	0.01 (0.00 to 0.02)	0.008
Diagnosed mental illness (not serious mental illness) × WEMWBS score	–	–	0.02 (0.01 to 0.02)	<0.001	0.01 (0.01 to 0.02)	<0.001
Diagnosed serious mental illness × WEMWBS score	–	–	0.04 (0.03 to 0.05)	<0.001	0.03 (0.02 to 0.04)	<0.001
Covariates						
Age, years	–	–	–	–	−0.01 (−0.01 to −0.01)	<0.001
Male versus female	–	–	–	–	−0.01 (−0.05 to 0.04)	0.792
White (reference)	–	–	–	–	0	−
Black	–	–	–	–	−0.08 (−0.23 to 0.07)	0.312
Asian	–	–	–	–	−0.08 (−0.18 to 0.01)	0.086
Others	–	–	–	–	−0.20 (−0.36 to −0.03)	0.019
Number of categories of long-standing physical illnesses	–	–	–	–	−0.33 (−0.35 to −0.31)	<0.001
GHQ (6-item score)	–	–	–	–	−0.02 (−0.03 to −0.01)	<0.001

WEMWBS, Warwick-Edinburgh Mental Well-Being Scale; GHQ, General Health Questionnaire.

**Fig. 2:**
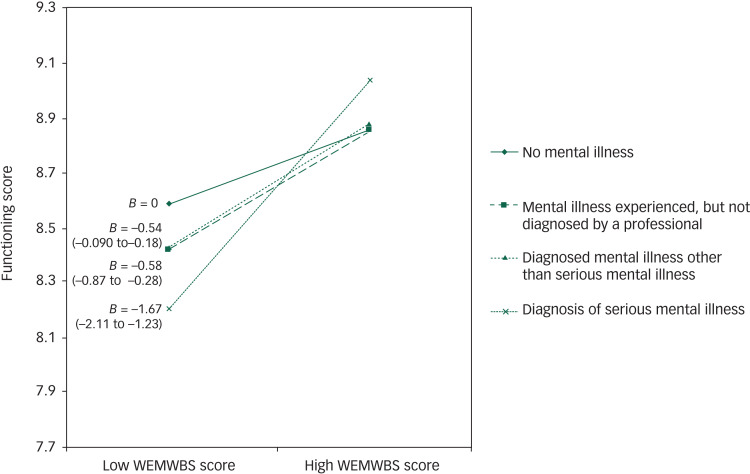
Graph showing gradients of the association between mental well-being and functioning scores by group after inclusion of covariates (model 3).

In Fig. 1, prior to including covariates, the steepest gradient (between mental well-being and functioning) was seen for the serious mental illness group, indicating the largest difference in functioning scores between those with high and low WEMWBS scores. Despite the gradients of all slopes decreasing between model 2 and model 3, the gradient for the serious mental illness group remained different from the other three groups to a statistically significant degree (Fig. 2). After adjusting for covariates in model 3, the effect size for the interaction between mental illness group and mental well-being, Cohen's *f*^2^, was reduced by 14.1% to 0.009.

*B* (95% CI) labelled is the regression coefficient.

## Discussion

### Main findings

Mental health is a major concern for all governments and healthcare systems, being inextricably associated with economic productivity, physical health and demand on health services. The promotion of mental well-being is a key public policy in many countries. Likewise, promoting recovery from mental illness has important personal, clinical, economic and other societal benefits. There is, however, a dearth of evidence directly linking mental well-being to improved functional outcomes and recovery from episodes of mental illness.

We found that social functioning varied with mental well-being, and that this association was modified by self-reported history of mental health problems to a statistically significant extent. Crucially, we found that the association between (greater) mental well-being and (better) functioning was greatest for respondents who reported having a diagnosis of serious mental illness, which included conditions such as schizophrenia or bipolar disorder. This interaction between mental well-being (WEMWBS score) and mental illness group in the association with function scores was statistically significant before and after adjusting for a range of potential confounders. In fact, including these covariates in the model seemed to further differentiate the diagnosed serious mental illness group from the other three groups, as seen in Figs 1 and 2, arguing against this association being artefactual. This suggests, albeit in a tentative and preliminary way, that improving mental well-being might support improvements in functioning (and recovery) in those with serious mental illness.

Slightly less than one-quarter of those within the group with diagnosed serious mental illness had a mental well-being score above or equal to the median score of the group with no mental illness. This finding, albeit cross-sectional and descriptive, supports the view that mental well-being can persist despite serious mental illness,^43^ and that there is a group of people with diagnosed serious mental illness who enjoy a high level of mental well-being. Further research on therapeutic targets might benefit from better understanding how to enhance mental well-being in people who experience episodes of serious mental illness.

### Strengths and limitations

HSE 2014 comprises a large and nationally representative sample of adults living in private households in England, and includes reliable and valid measures of health status. Those living in nursing and care homes were not included, although only a very small minority of people with the most severe and enduring mental illnesses reside in such settings. The main limitation of this study was its cross-sectional design, which precludes determination of the direction of causality. It is possible, of course, that functional recovery leads to greater mental well-being. Although the range and quality of data on potential confounders (including equivalised household income, physical illness and GHQ-12 scores) was a strength, we cannot exclude the possibility that our findings were the result of residual confounding. We did not, for example, have data on diagnosis, duration of mental illness or history of service use.

Functioning was assessed using the EQ-5D. Although this is a widely used and validated measure of functioning, it does not capture in-depth data about individual social activities. As certain social activities (such as employment) may differ between serious mental illness and non-serious mental illness groups, it was valuable that the EQ-5D ‘usual activities’ item asks about difficulty undertaking activities that are usual for each person rather than those they might not normally do.

The self-reported nature of our exposure variable (mental illness status) may have been prone to misclassification. However, any tendency to underreport more serious conditions (perhaps because of the perceived stigma associated with mental illness) is likely to have biased our findings (and differences between groups in functioning score) towards the null. In considering the role of chance, we note that the sample size of the diagnosed serious mental illness group was smaller (*n* = 241) than other exposure groups. For one, since respondents in the serious mental illness group reported the largest number of long-standing physical illnesses, we cannot exclude the possibility of residual confounding arising from the HSE method of recording only six conditions. However, given that we controlled for substantial variation in physical health between groups, it is unlikely that including an additional small number of uncounted conditions would have altered our main findings significantly.

Such variation in sample sizes between groups is not unusual in observational research. This also resulted in differences between groups in the width of CI around estimates of mean WEMWBS and functioning scores. Hence, 95% CI were presented to be cognisant of sample size variations. In addition, the risk of type II error was present, particularly in respect of tests for interactions between well-being and mental illness group. It is unlikely that our results were due to type I error, and we were careful to reduce the number of statistical tests undertaken.

While this study did not set out to formally test whether mental well-being and mental illness are independent of one another, our ultimate aim was to understand if, in principle, interventions to improve mental well-being (separate from treating the symptoms of mental illness) might have potential to support and enhance functional recovery. These findings need to be replicated in independent samples and in longitudinal and interventional research. If, indeed, further evidence is found that mental well-being improves functioning in people with mental illness, this could have profound policy and service implications. Since functioning is integral to recovery in mental illness, commissioners and providers would need to consider how best to deliver services that enhance mental well-being as well as symptom resolution. Such findings might also prove important in informing the treatment and care of people with mental illness for whom symptoms do not remit in response to treatment.

### Implications

Mental well-being was associated with better functioning, and this association was most evident in those who reported having received a diagnosis of serious mental illness. Even after adjusting for covariates, the plots showed the steepest gradient in the association between mental well-being and functioning for this serious mental illness group. The findings from this study were limited mainly because of the use of secondary cross-sectional survey data. Other longitudinal or intervention study designs could be considered in future research to explore causality and potentially revise treatment targets within health policies. If confirmed, these findings suggest that interventions to improve mental well-being could enhance recovery outcomes in people with serious mental illness.

## Data Availability

The data that support the findings of this study are available upon request to the UK Data Service at https://beta.ukdataservice.ac.uk/datacatalogue/series/series?id=2000021#!#format.
